# Observations on the Use of Zincum Vitriolatum

**Published:** 1803-01-01

**Authors:** Elijah Impey

**Affiliations:** Surgeon, Royal Navy, Member of the Royal College of Surgeons, London


### *Observations on the Use of Zincum Vitriolatum; communicated by Mr.* Elijah Impey, *Surgeon, Royal Navy, Member of the Royal College of Surgeons, London.*

WHILST our Navy continued off the coast of Egypt, many cases of dysentery occurred on board the ship I was surgeon of, in the primary stages of which there did not occur any peculiar symptoms or method of treatment to merit remark: Tenesmus, griping, discharge of mucus mixed with blood, and hardened scybalæ were removed by laxatives, enemas, diluent mucilaginous drinks, with attention to cleanliness and regimen; after which there existed an extreme degree of atony, and frequent discharge of fæces without pain. To obviate the latter, tonics, corroborants, and astringents were assiduously administered; in the list of which, I beg leave to assure you, there did not appear any medicine of such singular utility and efficacy as the zincum vitriolatum combined with opium.

*Edinburgh, Dec.* 1, 1802.

